# Prevalence of lumpy skin disease and associated risk factors in beef cattle in Rembang Regency, Central Java, Indonesia

**DOI:** 10.14202/vetworld.2025.76-84

**Published:** 2025-01-14

**Authors:** Yayan Taufiq Hidayat, Roza Azizah Primatika, Yatri Drastini

**Affiliations:** 1 Veterinary Science Study Program, Faculty of Veterinary Medicine, Universitas Gadjah Mada, Yogyakarta, Indonesia; 2 Animal, Fish and Plant Quarantine Center, Indonesian Quarantine Authority, South Papua, Indonesia; 3 Department of Veterinary Public Health, Faculty of Veterinary Medicine, Universitas Gadjah Mada, Yogyakarta, Indonesia

**Keywords:** lumpy skin disease, prevalence, risk factors

## Abstract

**Background and Aim:**

Lumpy skin disease (LSD) is an economically devastating infectious disease in cattle. Rembang Regency, located in Central Java, Indonesia, has suffered over 3800 cases of LSD and 75 deaths since early 2023. This region holds the 4^th^ number of most populous beef cattle producers in Central Java. However, until now, there have been no reports on the prevalence and risk factors related to LSD in beef cattle in Rembang Regency, Central Java, Indonesia. Therefore, this study aimed to estimate the prevalence of LSD and identify associated risk factors in Rembang Regency, Central Java, Indonesia.

**Materials and Methods:**

The sample size was 458 cattle, which were determined using the formula (n = 4PQ/L^2^) and two-stage random sampling technique, were examined physically through LSD typical clinical signs, namely distinguishing firm, circumscribed, few (mild forms) to multiple (severe forms) skin nodules. Structured questionnaires and interviews with farm owners were used to identify risk factors. The data related to the LSD were analyzed using descriptive statistics, bivariate analysis with Chi-square and odd ratios, and multivariate logistic regression to retrieve the logit model. All data were compiled in Microsoft^®^ Excel, while analyses were performed using SPSS version 26.0.

**Results:**

The prevalence of LSD in Rembang Regency was 28.2%. There were 11 significant risk factors associated with LSD. The multivariate analysis indicated that risk factors significantly contributing to LSD were knowledge of LSD transmission (p = 0.035, Odds ratios [OR] = 2.933), waste management (p = 0.014, OR = 4.015), rainy season (p = 0.019, OR = 2.944), and proximity between farms (p = 0.003, OR = 4.506). The logistic regression model analysis was as follows: LSD (Y) = −6.719 + 1.041 (knowledge of LSD transmission) + 1.390 (waste management) + 1.080 (rainy season) + 1.505 (proximity between farms).

**Conclusion:**

This study revealed a significant prevalence of LSD in Rembang Regency, Central Java, Indonesia, with 28.2% of cattle affected. Key risk factors contributing to LSD outbreaks were insufficient knowledge of transmission, inadequate waste management, seasonal rainfall, and close proximity between farms. These findings emphasize the need for targeted interventions, including educational programs for farm owners, improved waste management practices, and strategies to mitigate seasonal and spatial risks to control LSD in the region effectively.

## INTRODUCTION

Lumpy skin disease (LSD) is an economically important, transboundary, infectious disease in cattle caused by the LSD virus [1–3]. The virus belongs to the genus Capripoxvirus, family Poxviridae, and is a prototype strain of Neethling virus [4]. LSD is highly host-specific in cattle and water buffalo [5], although some wild ruminants are also susceptible to experimental conditions, including giraffes and impala [6]. The disease is clinically characterized by superficial nodules that are firm, round, raised, few to large numbers, and 2–5 cm in diameter [7]. The other clinical signs are high fever, swollen lymph nodes, increased nasal secretion, salivation, lacrimation, lameness, and edematous swelling in the abdomen, perineum, and limb region [8].

Transmission occurs through blood-sucking arthropods, mainly mosquitoes, stable flies, and ticks, as mechanical vectors [1]. Direct transmission is possible through nodular lesions, milk, saliva, and lacrimation by sharing feeding and watering ponds, although it is not significant compared with vector transmission [2, 9]. The disease has high morbidity ranging from 3% to 85%, with mortality >10% [3]. Despite its low mortality, LSDs may cause a devastating economic burden due to decreasing milk production, inappetence, lack of draft power, weight loss, abortion, and even death [8]. Therefore, LSD is classified as a notifiable disease by the World Organization for Animal Health [1].

The first LSD was documented in Zambia in 1929. Initially, LSD was thought to be limited to Africa, but it spreads rapidly in the cattle population in 1988–1989 in Europe and 1990 in the Middle East. The disease emerged in Southeast Asia in 2019, and it spreads widely in Southeast Asia in 2020 [10]. In 2022, LSD was reported for the 1^st^ time in Indonesia and spread across Sumatra and Java. Until May 2023, the disease had been confirmed in 15 provinces across Sumatra, Java, and Kalimantan islands [11].

Rembang Regency, located in Central Java, ranks 4^th^ populous beef cattle in the province, making it one of the cattle beef suppliers. The LSD hits Rembang Regency for the 1^st^ time in early 2023, causing serious problems, especially for small-backyard farmers, due to mortality and expenses of care and control. Most farmers are smallholders, with only 3–4 beef cattle maintained in the house’s backyard. Just like most farmers in Indonesia, cattle are not the main source of family income; rather, they are reared as savings, which will be sold as if there’s an urgent need [12, 13]. Nonetheless, cattle play an important role in the family economy because average farmers are categorized as having low-to-moderate incomes. As with many traditional cattle farming systems in Rembang Regency, low biosecurity measures and poor awareness may lead to disease outbreaks. Furthermore, Rembang Regency is located at a low altitude with a dry tropical climate, and it is strategically surpassed by the national north coast road (Pantura), which will possibly become the main livestock traffic route between the provinces of Java. The total number of LSD cases in 2023 was 3,800 infected cattle, of which 75 died, according to the veterinary authority.

Therefore, this study aimed to estimate the prevalence of LSD and identify associated risk factors in beef cattle in Rembang Regency, Indonesia.

## MATERIALS AND METHODS

### Ethical approval and Informed consent

This study was approved by the Animal Ethics Committee of the Faculty of Veterinary Medicine, Gadjah Mada University (101/EC-FKH/Int./2023, dated September 21^st^, 2023). Verbal consent was obtained from the participants before the interview.

### Study period and location

**Figure 1:**
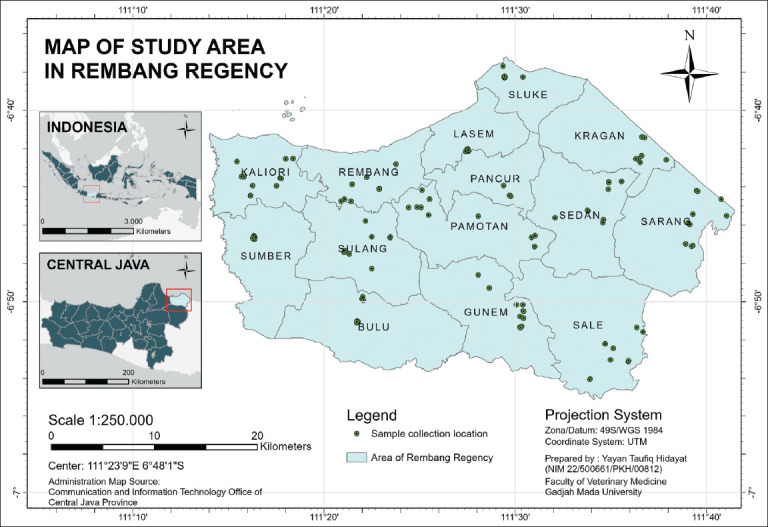
The study area of Rembang Regency, Central Java, Indonesia (Source: ArcGIS PRO ESRI^®^, USA).

The study was conducted from October 2023 to December 2023 in Rembang Regency, Central Java, Indonesia (6.8082°S, 111.4276°E). The study area consisted of 14 districts (Figure 1), and samples were collected using a proportional, simple random sampling method.

### Study design and sample size

A cross-sectional study with a two-stage random sampling method was used in this research. The sample size was determined based on the beef cattle population because of the lack of data on the number of farmers in Rembang Regency. The samples were counted using the formula n = 4PQ/L^2^ (n = sample size of the cattle sample, P = assumed prevalence using 4.17% reported in Thailand [10], Q = 1−P, L^2^ = desired confidence level 95%) [14]. The sample size was multiplied by five to avoid bias (n = 64 × 5 = 320 cows). Assuming a farmer owns three cows, the farmer’s sample was determined to be 107 persons (n = 320/3).

### Data collection

Data on the beef cattle population were obtained from the Department of Agriculture and Food in Rembang Regency. LSD was diagnosed based on the characteristics of clinical symptoms and confirmed by PCR testing of skin nodules (2–5 cm in diameter) or the condition named *sit-fast*, which formed from larger lesions and slough off and serves as the nidus for vector attraction and secondary bacterial infection [15]. The other clinical signs were fever, inappetence, lethargy, and lameness. Information related to risk factors was obtained from an interview with the farm owner who was chosen as the respondent. All data were analyzed using Microsoft^®^ Excel 365 (Microsoft Corporation, Washington, USA), SPSS Version 26.0 (IBM Corp., NY, USA), and ArcGIS Pro (ESRI^®^, USA).

### Questionnaire

Primary data on the risk factors of LSD were obtained by interviewing farmers using a questionnaire. Before use as a research instrument, the questionnaire was pretested for validity and reliability using the Statistical Package for the Social Sciences Version 26.0 (SPSS) (IBM Corp.). The questionnaire was administered to 20 respondents. The questionnaire items were considered valid when Pearson correlation had a p < 0.05 and reliable when Cronbach’s Alpha had a value >0.6.

### Statistical analysis

Descriptive, bivariate, and multivariate analyses were performed using SPSS version 26.0 (IBM Corp., USA). Univariate analysis was performed on the frequency of LSD (dependent variable) and the frequency of risk factors (independent variable). In the bivariate analysis, the Chi-square tests and p-values were used to identify whether the relationship between the risk factors on LSD is significant or not. The Chi-Square determined independent variables that meet the criteria for the basic model (p < 0.25) with a 95% confidence level and 5% desired error. Odds ratios (OR) are then considered when there is a significant relationship between LSD and risk factors to observe the association strength. The first step of the analysis was to develop the basic bivariate analysis model. Variables that were not p < 0.05 were gradually removed. A confounding test was performed if a variable could change OR >10%. The final step of the multivariable analysis was the development of a logistic regression analysis.

## RESULTS

A total of 458 cattle owned by 138 farmers were physically examined for LSD. The Sedan district had the highest sample proportion, with 50 cattle and 12 respondents. The prevalence of LSD in each district and the risk factors are presented in Tables 1 and 2. The prevalence of LSD in beef cattle in Rembang Regency was 28.2%. The Chi-square test of a 2 × 2 table (dichotomy) in the bivariate analysis showed the relationship between the dependent and independent variables. The results indicate that some factors, including monthly income, knowledge of LSD transmission, farming system, ventilation, vector eradication, farm cleaning, season of occurrence, distance between farms, quarantine of sick animals, and fences surrounding farms, have a significant relationship with LSD. The final multivariate analysis indicated that knowing the LSD transmission, waste management, season, and distance between farms had a truly significant relationship with LSD (Table 3).

**Table 1:** The prevalence of lumpy skin disease in beef cattle in Rembang Regency, Indonesia.

Number	Districts	Number of cattle samples	Infected cattle	Prevalence (%)
1	Bulu	31	10	32.3
2	Gunem	36	10	27.8
3	Kaliori	39	12	30.8
4	Kragan	27	9	33.3
5	Lasem	15	6	40.0
6	Pamotan	36	8	22.2
7	Pancur	20	4	20.0
8	Rembang	27	10	37.0
9	Sale	48	18	37.5
10	Sarang	35	3	8.6
11	Sedan	50	19	38.0
12	Sluke	28	8	28.6
13	Sulang	25	1	4.0
14	Sumber	41	11	26.8
	Total	458	129	28.2

**Table 2:** Univariate and bivariate analysis of the LSD risk factors at the farm level.

Number	Variable	Categories	Univariate				Bivariate		
	
			LSD on farm				*χ* ^2^	p-value	OR

			Positive	Negative	Total	%			
1	Farmer’s age	<60 years	68	37	105	76.1	0.040	0.841	-
		≥60 years	22	11	33	23.9			
2	Education	<Senior high school	62	31	93	67.4	0.264	0.607	-
		≥Senior high school	28	17	45	32.6			
3	Farming experience	<15 years	27	13	40	29.0	0.129	0.719	-
		≥15 years	63	35	98	71.0			
4	Average monthly income	<IDR 3 million	76	35	111	80.4	2.643	0.104	2.016
		≥IDR 3 million	14	13	27	19.6			
5	To know LSD transmission	No	53	15	68	49.3	9.567	0.002	3.151
		Yes	37	33	70	50.7			
6	Number of cattle	≤3	56	21	77	55.8	0.466	0.495	-
		>3	34	27	61	44.2			
7	Farming system	Non-intensive	10	37	47	34.1	3.382	0.066	0.420
		Intensive	80	11	91	65.9			
8	Farm location	<200 m	82	5	87	63.0	0.086	0.770	-
		≥200 m	8	43	51	37.0			
9	Farm location near water bodies (e.g., river, lake, airways)	Yes	27	37	64	46.4	0.787	0.375	-
		No	63	11	74	53.6			
10	Sunlight or air ventilation may enter the farm	Bad	43	32	75	54.3	2.669	0.102	1.830
		Good	47	16	63	45.7			
11	Vector control	No	57	24	81	58.7	2.296	0.130	1.727
		Yes	33	24	57	41.3			
12	Disinfection in farm	No	74	11	85	61.6	0.525	0.469	-
		Yes	16	37	53	38.4			
13	Farm cleaning	Poor	61	34	95	68.8	18.810	0.000	5.108
		Good	29	14	43	31.2			
14	Waste management (manure or leftover feed)	Not well managed	56	37	93	67.4	19.361	0.000	5.540
		Well managed	34	11	45	32.6			
15	Season occurrence of the disease	Rainy	54	32	86	62.3	8.906	0.003	3.000
		Dry	36	16	52	37.7			
16	Distance between farms	Close (<15 m)	65	30	95	68.8	15.745	0.000	4.333
		Far (≥15 m)	25	18	43	31.2			
17	Introduction new cattle	Yes	32	33	65	47.1	0.258	0.611	-
		No	58	15	73	52.9			
18	Quarantine sick animal	No	81	15	96	69.6	9.839	0.002	4.091
		Yes	9	33	42	30.4			
19	Farm location close to road lanes	Near (<5 km)	24	35	59	42.8	0.003	0.958	-
		Far (≥5 km)	66	13	79	57.2			
20	Fences surrounding the farm	No	25	26	51	37.0	4.544	0.033	0.455
		Yes	65	22	87	63.0			

LSD=Lumpy skin disease, OR=Odds ratio

**Table 3:** Bivariate analysis between LSD occurrence and its risk factors with p *<* 0.25 in beef cattle in Rembang Regency, Indonesia.

Variables	p-value	Decision
Farmer’s age	0.841	Excluded
Education	0.607	Excluded
Farming experience	0.719	Excluded
Average income monthly income	0.104	Possibly included
Knowing about LSD transmission	0.002	Possibly included
Number of cattle	0.495	Excluded
Farming system	0.066	Possibly included
Farm location	0.770	Excluded
Proximity to water bodies (e.g. river, lake, waterways)	0.375	Excluded
Sunlight or air ventilation may enter the farm	0.102	Possibly included
Vector control	0.130	Possibly included
Disinfection on the farm	0.469	Excluded
Farm cleaning	0.000	Possibly included
Waste management (manure or leftover feed)	0.000	Possibly included
Season occurrence of the disease	0.003	Possibly included
Proximity between farms	0.000	Possibly included
Introduction new cattle	0.611	Excluded
Quarantine sick animal	0.002	Possibly included
Farm location close to road lanes	0.958	Excluded
Fences surrounding the farm	0.033	Possibly included

LSD=Lumpy skin disease

### Description of farms with LSD risk factors

Most of the farmers in Rembang Regency traditionally reared the cattle in the house’s backyard. The aim of owning cattle was to provide a side job apart from farming and family savings [13]. The farmers were mostly under 60 years old (76.1%) and the minimum education was high school (45.9%). Most farmers have an average monthly income of less than IDR 3 million (± USD 200) (80.4%) and know about LSD transmission (50.7%). The farming system is mostly intensive (65.9%), and it is located in areas <200 m (63%) away from water bodies, including rivers, lakes, and waterways (53.6%). Vector control was not applied at the farms (58.7%), while sanitation (68.8%) and waste management (67.4%) were poor. Disease occurred during the rainy season (62.3%). The farmer neither introduced new animals to the farm (52.9%) nor quarantined sick animals in a separate location (69.6%). Most farms are far from road lanes (57.2%), and there are fences surrounding the farms (63%).

### Bivariate analysis of LSD risk factors at the farming level

The bivariate analysis revealed that the significant risk factors are average monthly income which is less than IDR 3 million (p = 0.104, OR = 2.016), not knowing LSD transmission (p = 0.002, OR = 2.016), intensive farming systems (p = 0.066, OR = 0.420), the presence of ventilation that allows sunlight to enter the farm (p = 0.102, OR = 1.830), vector control applied in the farm (p = 0.130, OR = 1.727), poorly farm cleaning (p = 0.000, OR = 5.108), poorly manure and leftover management (p = 0.000, OR = 5.540), rainy season (p = 0.003, OR = 3.000), close distance between farms (p = 0.000, OR = 4.333), not doing quarantine of sick animals (p = 0.002, OR = 4.091), and no fence surrounding farms (p = 0.033, OR = 0.455). Meanwhile, the risk factors that were not significantly associated with the occurrence of LSD in Rembang Regency were farmer age (p = 0.841), education below senior high school (p = 0.607), farming experience <15 years (p = 0.719), number of cattle (p = 0.495), farm location (p = 0.770), near location to the river (p = 0.375), disinfection (p = 0.469), and introduction of new cattle to a new farm (p = 0.611).

### Multivariate analysis of LSD risk factors

The multivariate analysis obtained from logistic regression analysis indicated that the LSD model from this analysis is LSD (Y) = −6.719 + 1.041 (knowing LSD transmission) + 1.390 (waste management) + 1.080 (rainy season) + 1.505 (distance between farm). The results of logistic regression analysis of the risk factors of LSD indicated that the factors contributing to the increased occurrence of LSD in livestock were lack of information due to LSD transmission (β = 1.041, OR = 2.833), poor waste management (β = 1.390, OR = 4.015), rainy season (β = 1.080, OR = 2.944), and close distance between farms (β = 1.505, OR = 4.506) (Table 4). The model was relatively accurate as it passed the goodness-of-fit (Hosmer-Lemeshow) test with a sensitivity of 77.1% and specificity of 74.4%.

**Table 4:** Multiple logistic regression analysis of risk factors for LSD in beef cattle in Rembang Regency, Indonesia.

Variables	Coefficient (β)	p-value	OR	95% CI
Knowing about LSD transmission	1.041	0.035	2.833	1.073–7.480
Waste management	1.390	0.014	4.015	1.330–12.123
Rainy season	1.080	0.019	2.944	1.196–7.244
Proximity between farms	1.505	0.003	4.506	1.678–12.104
Constanta	−6.719	0.003		

LSD=Lumpy skin disease, OR=Odds ratio, CI=Confidence interval

## DISCUSSION

There have been no prevalence studies on LSD in Indonesia, but the prevalence of LSD in beef cattle in Rembang Regency is 28.2%. The prevalence of LSD in this study was higher than that of LSD in Natore, Bangladesh [16], Ethiopia [17], and Egypt [18], but it is smaller than LSD prevalence in Naogaon [19] and Dinajpur Sadar, Bangladesh [20]. Based on the district area, the highest prevalence of LSD in beef cattle was found in the Lasem district at 40%, whereas the lowest prevalence was found in the Sulang district at 4%. During the observation during the research, many sheds were in poor condition of hygiene and sanitation with an open-space cattle dung dump near the pen. Aside from the cattle barn being in the backyard of the house and due to the dense population, the proximity between cow pens cannot be avoided. These factors may increase the abundance of vectors that contribute to disease occurrence [2]. The prevalence of the disease varies across different regions due to several factors, such as geographic distribution, the abundance of the vectors that can influence the spread of the disease, herd size, vaccination program, the availability of resources for disease control, and the level of awareness among farmers [6, 10, 21, 22].

The significant risk factors for LSD in livestock farms based on bivariate analysis were average monthly income of less than IDR 3 million, lack of information on LSD transmission, non-intensive farming system, bad ventilation that may sunlight enter the farm, no vector control, poor farm cleaning and waste management, rainy season, close distance between farms, no quarantine for sick animals in separated places, and no fences surrounding the farm. Farmers with low incomes (less than IDR 3 million) had significant values of p = 0.104 and OR 2.463. The results indicated that low-income farmers have a 2.463 times higher risk for LSD than those with higher monthly incomes (more than 3 IDR 3 million). LSDs have become an important concern for low-income farmers, particularly in regions where livestock plays a crucial role in sustaining livelihoods. Low-income farmers may not afford the medication for their cattle and implement biosecurity measures, including proper fencing, quarantine facilities, and regular cleaning [23, 24].

Lack of information related to LSD transmission had a significant value of p = 0.002 and OR = 9.567. This suggests that the lack of information related to LSDs is 9.567 times higher than that of farmers with knowledge about LSDs. A farmer’s knowledge and perception of the LSD are the most important factor in the prevention and control of the disease [25]. Without knowledge of the symptoms and transmission of LSD, farmers might not recognize early signs of the disease, which could delay diagnosis and treatment [26]. Intensive awareness campaigns, such as social media, print, talks, and training programs, are some actions that are needed to increase farmers’ knowledge [27]. Veterinarians and animal health officers in Rembang Regency took action in the hope of mitigating the spread of the disease and increasing farmers’ awareness.

The risk factor for the extensive farming system had a significant value of p = 0.066 and OR = 0.420, indicating that extensive farming was associated 0.420 times with LSD occurrence. Extensive farming systems can significantly increase exposure to biting insects, such as mosquitoes, flies, and ticks, which are the primary vectors of LSD. The lack of a controlled environment increases the likelihood of the vector to contact with a susceptible animal [25, 28]. Another risk factor was the presence of ventilation so that sunlight and airflow could enter the shed. It had an association of p = 0.102 and OR = 1.830, which means poor ventilation and ventilation might increase the LSD by 1.830, which is higher than that for a shed with good sunlight and airflow ventilation. Poor ventilation and circulation in barns and shelters, which are characterized by inadequate sunlight and airflow, can cause high humidity in farms. This environment creates a favorable environment for the breeding and survival of insects, such as mosquitoes and stable flies, which are primary vectors of LSD. The LSD virus can survive in scabs and the environment for up to 6 months under protected sunlight conditions [22].

Vector control in farms was associated with p = 0.130 and OR = 1.727, indicating that farms with no vector control were 1.727 times more predisposed to LSD than those with an adequate control vector. The control included fumigation or burning waste (traditional method) at night to reduce mosquitoes or spraying insecticides to control flies, mosquitoes, and ticks [29]. As transmission occurs by blood-sucking arthropods, such as mosquitos, flies, and ticks, vector profusion in farms can increase the risk of LSD occurrence [1]. Thus, vector control was necessary on the farm.

Sanitation on the farm had a significant association with p = 0.000 and OR = 5.108, indicating that farms with poor sanitation had 5.108 times higher than farms with good sanitation. Poor sanitation can accumulate stagnant water and organic waste, which serve as breeding grounds for mosquitoes and flies. It also contributed to a higher number of ticks. Vectors thrive in unclean environments, and when hygiene is poor, an increased vector population can lead to a higher rate of LSD transmission [30, 31]. Furthermore, farms that did not manage animal waste properly were significantly associated with LSD (p = 0.000 and OR = 5.540). The results indicated that farms with poor waste management were 5.540 times more likely to have LSD than those with good waste management. This may be related to vector abundance on the farm. Vectors prefer wet areas with scattered or piled feces on farms. The leftover feed can be burned, urine can be collected and used as fertilizer, and feces can be used as compost. Feces should be piled up in animal pens or in the farm area but should be placed in a covered area far away from the farm. Proper waste management can reduce vector breeding sites on farms [29].

The rainy season had a significant association with LSD ([p = 0.003] and [OR = 3]), indicating that LSD was 3 times higher when it occurred in the rainy season than when it occurred in the dry season. The rainy season provided ideal conditions for the multiplication and increased activity of vectors, which facilitated the spread of the disease [2]. Wet and increased humidity during the rainy season created an ideal environment for vector growth and proliferation [15]. The proximity between farms had a significant association with LSD (p = 0.000 and OR = 4.333). The results indicated that close farms (<15 m) were 4.333 higher than the farms with far distances in between. Higher farm density may lead to an overall increase in the vector population due to more breeding sites and hosts in a concentrated area. Most farmers in Rembang Regency kept their cattle in the house’s backyard. Vectors such as mosquitos and flies can easily travel between nearby farms, increasing the risk of LSD transmission. Furthermore, pathogens can spread more easily through vectors, contaminated equipment, and personnel moving between farms [29].

The biosecurity activity of quarantining sick animals to separate barns and the existence of fences surrounding farms were also significant risk factors for LSD. Farms without quarantined sick animals had a significant association with LSD (p = 0.002 and OR = 4.091), which means the activity might lead to LSD occurrence 4.091 more likely than those farmers who had quarantine facilities for separating sick animals. The farms without fences surrounding the farms had an association with LSD (p = 0.033 and OR = 0.455). The results indicated that farms without fences were prone to LSD 0.455 times higher than farms with fences. Sick animals, due to LSD infection, can easily transmit the virus to healthy animals through direct contact. Infected animals remain with the herd without quarantine, leading to rapid spread. Sick animals might also contaminate shared resources such as water, feed, and equipment, leading to infection among healthy animals. Fencing helps control who and what enters the farm. Without fences surrounding the farm, there was a higher risk of biosecurity breaches from people, animals, and equipment that might carry the LSD virus [32].

The regression model in this study was LSD (Y) = −6.719 + 1.041 (knowing LSD transmission) + 1.390 (waste management) + 1.080 (rainy season) + 1.505 (distance between farms). These four risk factors may contribute to and influence LSD occurrence in beef cattle in the Rembang Regency. The predictor variable of knowing LSD transmission had an OR of 2.833 (95% confidence interval [CI]: 1.073–7.480), indicating that a lack of knowledge about LSD transmission contributed 2.833 times more to disease occurrence than farmers with adequate knowledge. The waste management predictor gave an OR of 4.015 (95% CI: 1.330–12.123), indicating that poor waste management practices contributed to LSD 4.015 higher than those with good waste management practices. The rainy season had an OR of 2.944 (95% CI: 1.19 –7.244) which shows that LSD cases increase 2.944 times in rainy and wet conditions. Finally, proximity between farms had OR 4.506 (95% CI: 1.678–12.104). Close distance between farms (<15 m) contributed 4.506 times more than distances >15 m.

Therefore, control and prevention measures should be taken to anticipate the further spread of the disease. Effective campaigns and awareness are essential for educating farmers about LSD and promoting preventive measures, including good-practice waste management. Farmers must provide hygiene and shelter management practices during the rainy season to ensure water flow and anticipate the vector population. Ensuring access to veterinary care is also critical.

## CONCLUSION

The study has effectively identified the prevalence and critical risk factors for LSD in beef cattle in Rembang Regency, Central Java, Indonesia, with a reported prevalence of 28.2%. Key risk factors include a lack of knowledge about LSD transmission, poor waste management practices, heightened vector activity during the rainy season, and the close proximity of farms. These findings underscore the urgent need for targeted interventions to control LSD. Farmer education on disease transmission and prevention, combined with improvements in farm sanitation, biosecurity measures, and vector control, is essential to reduce the disease’s spread and impact on smallholder farmers who rely on cattle for income and savings.

Future efforts should focus on enhancing disease surveillance and mapping to identify high-risk regions and emerging patterns. Public awareness campaigns and farmer training programs should be prioritized to improve biosecurity and waste management practices. Research into sustainable vector control methods and the development of effective vaccines and treatments will be critical for long-term disease management. Strengthening veterinary support systems and policies to assist low-income farmers in adopting preventive measures can further mitigate the effects of LSD. In addition, investigating the impact of climatic factors on disease transmission can help develop adaptive strategies tailored to tropical and wet environments like those in Rembang Regency. These integrated approaches will contribute significantly to control LSD and ensure the sustainability of cattle farming in the region.

## AUTHORS’ CONTRIBUTIONS

RAP: Designed, conceptualized, and supervised the study and revised the manuscript. YTH: Collected and analyzed the data, methodology, data curation, investigation, and drafted the manuscript. YD: Supervised the study, validation, and methodology. All authors have read and approved the final manuscript.
